# We have a problem!

**DOI:** 10.4103/0971-3026.43832

**Published:** 2008-11

**Authors:** Bhavin Jankharia

**Affiliations:** Bhaveshwar Vihar, 383, Sarda V P Road, Mumbai – 400 004, India E-mail: editor@ijri.org

We have achieved many of the objectives that we set out to achieve: The journal now comes out on time. There is a defined structure. We have been successful, at least partly, in discouraging and thereby reducing the number of case reports. We now have regular features, pictorial essays, reviews, and editorials.

But we still lack original clinical research and outcome papers. Such articles make up less than 5% of the papers received; 75% of submissions are still case reports. 

It is not enough for the journal to serve as a continuing medical education (CME) tool. It also needs to showcase the practice patterns and the clinical research being done in radiology in this country.

The majority of the clinical research articles that we get are converted theses. These are usually badly written, with no understanding of statistics and no statistical relevance. They may get accepted by the Universities and by the National Board of Examinations, but that does not necessarily mean that these theses meet the journal's standards.

It is a chicken-and-egg situation. Since we are not indexed, good researchers do not want to send articles to the journal; and until we get more such articles, it is unlikely that we will get indexed.

Nevertheless, we need to find a solution and I solicit your suggestions and advise. Please email me at editor@ijri.org.

Some possible suggestions are:

1. All heads of departments need to constructively push their residents into planning clinical studies and writing them up. There is significant material available in this country.
2. The IJRI should try and hold ‘writing’ courses.
3. All studies presented in the annual conference may be fast-tracked.

This issue starts off with a symposium on obstetric USG that has been guest edited by Dr Ramamurthy and Dr Gune. There will be more articles on the same subject in the February 2009 issue, to be followed by a symposium on breast imaging in the May and August 2009 issues.

Dr N. Khandelwal's oration from 2008 on ‘CT Perfusion in Stroke’ has been published in this issue. Dr V. Rangarajan gives us his perspective on the current status and growth of PET/CT in India. We have reproduced two articles by Dr K. P. Mody, from the IJR issues of the mid-50s, on radiology teaching and curriculum and ethics; they are relevant even today.

The response to the last issue's editorial has been quite heartening. One interesting quote: *‘Residents hardly take on any responsibilities. All the major decisions are taken by consultants. PGs are more or less doing an ‘observership’ kind of posting-which is extremely sad. As a result, we are churning out a bunch of spineless, gutless radiologists who need to spend at least 3 more years in a decent place to expose them to the real big bad world of radiology.’* It is quite clear on talking to a cross-section of teachers in the country that the current batch of residents and newly-minted radiologists are quite poorly trained, with no perspective on the clinical relevance of the various examinations. Most seem only interested in capitalizing on their education and maximizing their ‘earning’ potential, even if it is at the cost of their own growth. We need to remember that, unlike in other professions, radiologists tend to work till the ages of 70 and beyond, and we cannot sacrifice long-term benefits for the short-term.

In a subsequent editorial, I will show how even from an ‘earnings’ perspective a radiologist does much better than most MBAs when we compare apples to apples and oranges to oranges.

